# ﻿Two new species of *Szelenyiopria* Fabritius (Hymenoptera, Diapriidae), larval koinobiont endoparasitoids of the leaf-cutter ant *Acromyrmex coronatus* (Fabricius) (Hymenoptera, Formicidae), from Panama

**DOI:** 10.3897/zookeys.1250.151740

**Published:** 2025-09-01

**Authors:** Gabriel Araúz, Vasilisa Chemyreva, Tomás Ríos, Dumas Gálvez, Roberto A. Cambra, Enrique Medianero, William T. Wcislo, Hermógenes Fernández-Marín

**Affiliations:** 1 Centro de Biodiversidad y Descubrimiento de Drogas, Instituto de Investigaciones Científicas, Ciudad del Saber, Ciudad de Panamá, 0843-01103, Panama Centro de Biodiversidad y Descubrimiento de Drogas, Instituto de Investigaciones Científicas Ciudad de Panamá Panama; 2 Programa Centroamericano de Maestría en Entomología, Universidad de Panamá, Ciudad de Panamá, Panama Universidad de Panamá Ciudad de Panamá Panama; 3 Zoological Institute, Russian Academy of Sciences, 1 Universitetskaya Emb., St Petersburg 199034, Russia Zoological Institute, Russian Academy of Sciences St Petersburg Russia; 4 Museo de Peces y Agua Dulce (MUPADI), Universidad Autónoma de Chiriquí, Chiriquí, Panama Universidad Autónoma de Chiriquí Chiriquí Panama; 5 Coiba Scientific Station, City of Knowledge, Calle Gustavo Lara, Bld. 145B, Clayton, 0843-01853, Panama Smithsonian Tropical Research Institute Ancón Panama; 6 Sistema Nacional de Investigación, Panamá, Panama Sistema Nacional de Investigación Panamá Panama; 7 Smithsonian Tropical Research Institute, Apartado 0843, Balboa, Ancón, Panama Coiba Scientific Station Clayton Panama; 8 Museo de Invertebrados G. B. Fairchild, Escuela de Biología, Universidad de Panamá, Panamá, Panama Universidad de Panamá Panamá Panama

**Keywords:** Attini, endoparasitoid, fungus-growing ants, larvae, parasitism

## Abstract

Leaf-cutter ant nests attract a wide variety of guests and parasites due to considerable food resources, substrate and shelter available within nests. Limited information is known about their taxonomy, bionomics, and natural history, including interactions with the ants, and degree of host specificity of these symbionts. Here we describe two new species of *Szelenyiopria* Fabritius, 1974 (Diapriidae, Diapriinae, Diapriini) wasps which are koinobiont endoparasitoids of the larvae of a leaf-cutter ant, *Acromyrmex coronatus* (Fabricius, 1804) (Myrmicinae, Attini) in Chiriquí, Panama. For both new *Szelenyiopria* species, *S. fortunensis* Araúz & Fernández-Marín, **sp. nov.** and *S. chiriquiensis* Araúz & Fernández-Marín, **sp. nov.**, we provide information on parasite–host interactions. The most abundant and frequent parasitoid type was solitary, while gregarious parasitoidism, where two or more wasps emerged from a single ant larva, was associated with certain times of the year. We provide an updated key to females of all species of *Szelenyiopria*, their geographic distribution, and host–parasite interactions. *Szelenyiopria* previously had been identified as parasitoids of several ant genera including some *Acromyrmex* species, but our data indicate that some species of *Szelenyiopria* may be specialized on *A. coronatus* ants. Rates of parasitism suggest that the wasps have a negative effect on colonies and populations of the ants.

## ﻿Introduction

Ants are distributed nearly worldwide in diverse terrestrial ecosystems (Hölldobler and Wilson 1990; Savage et al. 2014). Their nests contain substantial amounts of food resources and individuals, which attract numerous natural enemies and cohabitants with different life strategies (Hölldobler and Wilson 1990; Hughes et al. 2002; Quevillon 2018). Among these, some parasitoid insects complete their life cycle within the nests of various ant species (Eggleton and Belshaw 1992; Lachaud and Pérez-Lachaud 2012). Parasitoids can enter the colony either directly, or by being transported by ants that are returning to the nest after foraging or other tasks (Wojcik 1989; De Bekker et al. 2018). Inside the colony, they deploy various strategies to evade the defensive responses of their hosts, including morphological and behavioral adaptations (McIver and Stonedahl 1993), or by mimicking the hydrocarbon chemical profiles of the host (Wojcik 1989; De Bekker et al. 2018; Neupert et al. 2018).

A little-known taxon of parasitoids that attacks ants is Diapriidae (Hymenoptera, Diaprioidea). They are koinobiont endoparasitoid wasps: the female injects an egg into the larva of the host ant where the wasp larva grows and feeds on internal tissues without consuming the cuticle, which becomes a protective cover for the parasitoid larva, analogous to a cocoon (Fernández-Marín et al. 2006; Loiácono et al. 2013). According to Paz et al. (2024), at least 30 genera of Diapriidae wasps are associated with ants primarily fungus-growing ants (Formicidae, Myrmicinae, Attini), including *Apterostigma* Mayr, *Acromyrmex* Mayr, *Cyphomyrmex* Mayr, and *Mycetomoellerius* Solomon, Rabeling, Sosa-Calvo & Schultz (formerly *Trachymyrmex*) (Fernández-Marín et al. 2006; Pérez-Ortega et al. 2010; Loiácono et al. 2013; Mattoso et al. 2021). The presence of these wasps in ant nests negatively affects ant colonies, resulting in considerable effect on their demographics, since wasps can parasitize up to 100% of the ant larvae (Fernández-Marín et al. 2006; Rodrigues et al. 2015).

Within Diapriidae, the genus *Szelenyiopria* Fabritius has 13 described species. Four species and two morphotypes are associated with ant species belonging to Attini and Ecitonini, including *Acromyrmex* (Loiácono 1987, 2000, 2013; Mattoso et al. 2021), Mycetomoellerius (Trachymyrmex) (Pérez-Ortega et al. 2010), *Eciton* Latreille (Ferrière 1929), and *Neivamyrmex* Borgmeier (Ferrière 1929). The host remains unknown for nine of the species described. In nearly all cases, very little is known about the biological interactions between *Szelenyiopria* and their hosts and its geographical distribution.

The aim of this work is (i) to describe two new species of *Szelenyiopria*, which are larval endoparasitoids of the leaf-cutter ant *Acromyrmex coronatus* in Panama; and (ii) to provide information about the biology of the parasitism and expand the knowledge of parasite–host interactions between *Szelenyiopria* wasps and fungus-growing ants.

## ﻿Materials and methods

### ﻿Ant hosts

**Figure 1.**
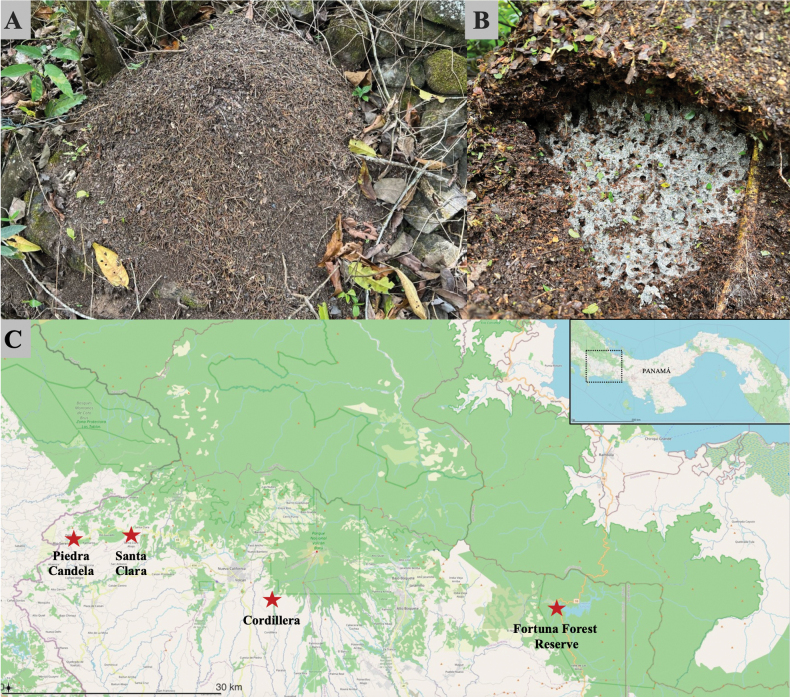
A. *Acromyrmex coronatus* nest covered with leaf litter; B. Nest with fungus garden exposed; C. map of the collection sites in Panama.

*Acromyrmex* is a genus of leaf-cutting ants comprised of 42 species, and *A. coronatus* has one of the wider geographical distributions. *Acromyrmex coronatus* has a Neotropical distribution inhabiting cloud and humid forests between 600 and 2,000 m of altitude (J. Longino in AntWeb 2024) from Mexico to Argentina, and the Atlantic Forest region of Brazil at sea level. The nests are found on the ground or among tree branches and contain fungus gardens covered with a dome of dry leaves and dry branches (Fig. 1A, B) (Gonçalves 1961). A mature colony has more than 150,000 individuals (Wetterer 1995), which is the largest colony size for any *Acromyrmex* species (Gonçalves 1961). Workers are of small to medium-sized (3–8 mm), and there are no soldier castes. Variation in biological characteristics of *A. coronatus* across its wide geographic range has not been studied. There are no distributional studies in Panama, but our observations suggest that this ant may be restricted to the highlands of the western region of Panama, at altitudes between 900 and 1900 m. Similar to other *Acromyrmex*, *A. coronatus* workers are parasitized by phorid flies (Phoridae), including *Apocephalus luteihalteratus* Borgmeier, *Ap. pseudocercus* Brown, and *Myrmosicarius catharinensis* Borgmeier (Quevillon 2018; Almeida et al. 2021). Parasitism of some *Acromyrmex* species by diapriid wasps has been reported previously (Loiácono et al. 2013; Mattoso et al. 2021), but there are no reports for *A. coronatus* as a host.

### ﻿Material studied

A total of 53 colonies of *A. coronatus* were collected in Chiriquí Province, Panama (Fig. 1C). Out of these, 32 nests were collected in the Fortuna Forest Reserve in 2012 and 2021 (8°43'21.1"N, 82°14'15.7"W); 13 were collected in Cordillera between 2021 and 2023 (8°42'33.0"N, 82°36'04.5"W); four nests were collected in Piedra Candela between 2021 and 2023 (8°52'51.3"N, 82°45'20.2"W); and four nests were collected in Santa Clara in 2021 (8°49'40.4"N, 82°43'49.0"W). All nests were between 900 and 1600 m a.s.l. The nests were identified by the presence of a litter dome that covers the fungus garden as reported by (Fernández et al. 2015). Additionally, the ants were identified by their occipital spines directed outwards and pronotal spines directed forward (Fernández et al. 2015). All nests were located by active searching during the day in the forest and along roadsides.

### ﻿Nest collecting

The litter dome was carefully removed, and we extracted the colonies as completely as possible, but some portions of the fungus garden were found between rootlets, which made it difficult to sample the entire garden. The sampling included most of the fungus garden, and host ants, including queens, workers, pupae, and larvae. Each fungus garden was placed in sterile 5 or 15 L plastic containers with wet KimWipes (Kimberly-Clark^®^) to maintain humidity. Since some ants were away foraging or came out to defend the nest during the nest collection, and we were unable to collect all the adult ants, so demographic data on adults may be underestimated (Pérez-Ortega et al. 2010).

Collected colonies were transported to the Social Insects Lab (LIS) at Instituto de Investigaciones Científicas y Servicios de Alta Tecnología (INDICASAT-AIP), Panama City, to determine whether the larvae were parasitized or not. Parasitized larvae were distinguished by the presence of brownish or blackish stains in their cuticle, while healthy larvae have a white or yellow coloration (Fernández-Marín et al. 2006; Pérez-Ortega et al. 2010; Loiácono et al. 2013; Mattoso et al. 2021).

To sample adult wasps, some parasitized larvae were isolated and placed in Petri dishes in the laboratory with a portion of the fungus garden, some ant workers, and wet KimWipes, with a mean temperature of 23 °C and humidity of 80%. Once the wasps emerged, they were collected to prevent the ants from cutting off their antennae, wings, and legs as reported by Fernández-Marín et al. (2006). Some wasps failed to eclose from the ant host, so those were dissected under a Zeiss Stemi 2000-C stereomicroscope to identify their sex and species.

### ﻿Morphological descriptions and taxonomic key

We followed Masner and García (2002) to identify the genus *Szelenyiopria*; both species were compared with other species of the genus based on the taxonomic descriptions of Fèrriere (1929), Loiácono (1987), Loiácono and Margaría (2000), Loiácono et al. (2013), and Comério et al. (2019). Additionally, we reviewed photographs of type material of *S. reichenspergeri* deposited at Muséum d’histoire naturelle, Geneva (**MHNG**), and type material of *S. clavata*, *S. coriacea*, *S. distinguenda*, *S. elongata*, *S. masneri*, *S. minima*, and *S. pilosa* deposited at the Canadian National Collection of Insects, Arachnids and Nematodes, Ottawa (**CNC**). We used Scanning Electron Microscope (**SEM**) images to visualize all the detailed characters of both species. Additionally, we utilized a Leica M205 C stereomicroscope adapted with Leica K|3C camera to measure the individuals. Terminology is based on Masner and García (2002). Types are deposited in the Museo de Invertebrados G.B. Fairchild at Universidad de Panama (**MIUP**) and LIS. The distribution map was created in R (R Core Team 2024).

### ﻿Parasitism

We recorded the overall parasitism rate (prevalence) of all the 53 nests, and the prevalence rates by species and sites. Prevalence is defined as: the percentage of parasitized nests out of the total nests collected. To understand how within-nest parasitism (intensity) impacts the colonies, we recorded the demography of 17 colonies collected in Cordillera and Piedra Candela by counting all eggs, healthy and parasitized larvae, pupae, workers, and queens. The within-nest parasitism rates (intensity) are defined as: the percentage of parasitized larvae out of the total larvae in the nest. Additionally, we recorded the solitary or gregarious parasitism rates, which are defined as the percentage of solitary or gregarious larvae out of the total parasitized larvae. Moreover, we estimated the time-scale parasitism, based on the month when parasitism was more frequent, and the sex ratio of emerged wasps in each nest. We only could calculate the prevalence for *S. fortunensis*. For *S. chiriquiensis* we calculated the prevalence, intensity, solitary or gregarious parasitism, time-scale parasitism, and the sex ratio.

### ﻿Data analysis

All analyses were performed in R (R Core Team 2024). We evaluated whether the number of parasitized larvae was influenced by the total number of eggs, number of larvae, season, and site. Total number of ants (all ages), number of workers and number of pupae were correlated with total number of larvae and were not included in the model. We first carried out generalized linear models (GLM) with Poisson and Quasipoisson distributions, but they showed significant overdispersion; therefore, we used a Negative Binomial Generalized Linear Model as implemented in the package MASS (function glm.nb).

## ﻿Results

### ﻿Taxonomic descriptions

#### ﻿ Szelenyiopria fortunensis

Taxon classificationAnimaliaHymenopteraDiapriidae

Araúz & Fernández-Marín sp. nov.

DD7A960D-B996-5338-9813-D96517509630

https://zoobank.org/C8792AFC-5916-4235-8949-F2D6AD6182A2

Figs 2, 3, 4, 5

##### Type material.

**Figure 2.**
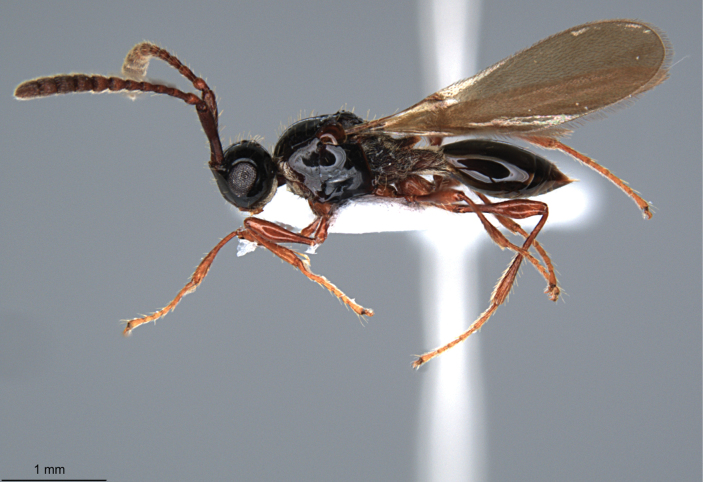
*Szelenyiopria fortunensis* sp. nov., holotype, female, habitus in lateral view.

***Holotype*** • female (Fig. 2). “PANAMÁ, Chiriquí, Gualaca, Reserva Forestal Fortuna (8°43'19.8"N, 82°14'14.8"W, 1200 m); 26.v.2012. Emerged from a nest of *Acromyrmex coronatus* (nest #25), Tomás Ríos, Juan Bernal and Hermógenes Fernández-Marín leg” (MIUP). ***Paratypes*** • with same data as holotype; 3 females and 1 male in MIUP; 3 females and 1 male in LIS.

##### Etymology.

The species is named after the Fortuna Forest Reserve, a protected area in the Chiriquí Province, where we found this species. Additionally, the species name refers to the fortunate and serendipitous encounter when one of the authors (HFM) discovered a nest of an *Acromyrmex* species parasitized by a diapriid wasp for the first time in Panama.

##### Diagnosis.

**Figure 3.**
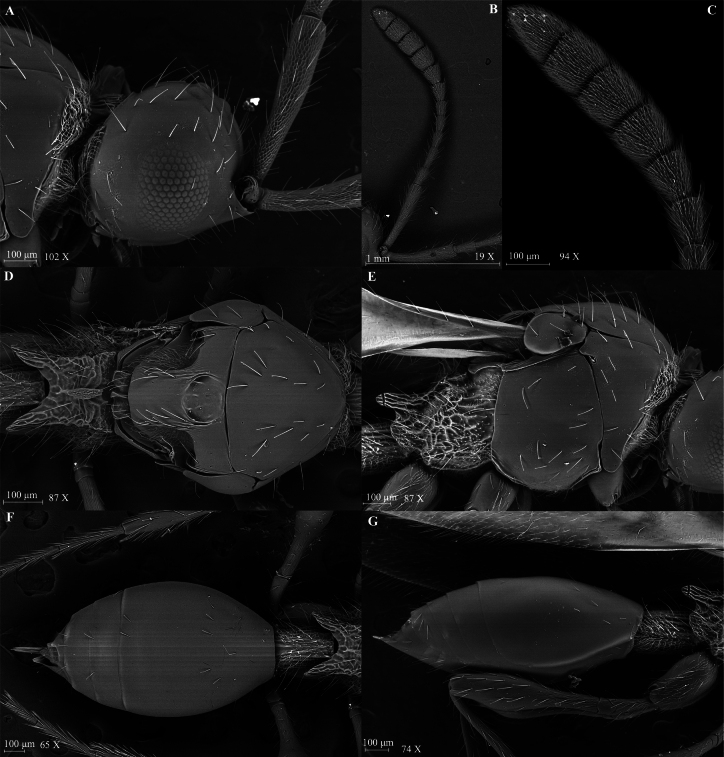
*Szelenyiopria fortunensis* sp. nov., female. A. Head in lateral view; B, C. Antennae in lateral view; D. Mesosoma in dorsal view; E. Mesosoma in lateral view ; F. Metasoma in dorsal view; G. Metasoma in lateral view.

**Figure 4.**
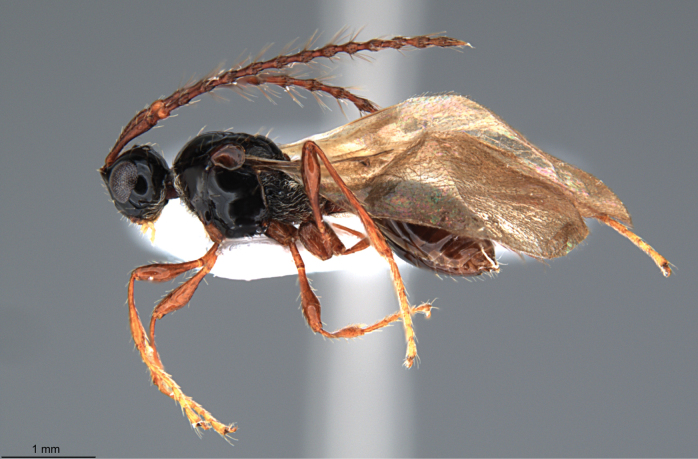
*Szelenyiopria fortunensis* sp. nov., paratype, male, habitus in lateral view.

*Szelenyiopria fortunensis* differs from the all known species of the genus by the combination of the following characters: female antenna 12-segmented with nonabrupt clava (Fig. 2B, C), four apical segments of clava flattened ventrally; gena with dense postgenal cushion; lateral side of pronotum and mesopleura smooth; anterior scutellar pit subcircular, smooth inside (Fig. 3D); mesoscutum mainly smooth; propodeum rugose sculptured with median keel strongly raised anteriorly to form “axe” shaped spine (Figs 1, 3E, 4D); sides of propodeum dorsally and metapleuron reticulate rugose sculptured with scattered, apically truncate and upstanding setae, and covered with short pubescence; T2–T4 covered with few setae (Fig. 2F, G). Males with 14-segmented antenna; A3–A14 each with a row of long setae apically.

##### Description.

Holotype, **female** (Fig. 2). Body length 3.31 mm; head, mesosoma and metasoma dark brown, smooth, and shiny; antenna, tegula, and legs pale brown.

***Head*** (Fig. 3A–C). Head subcircular, in dorsal view 1.03 × as wide as long (0.60 mm/0.58 mm) with protruding antennal shelf and covered with scattered truncate setae. Frons and face smooth. Temple length subequal to width of compound eye (0.21 mm/0.22 mm) in lateral view. Compound eyes ovoid, 0.73 × as wide as high (0.30 mm/0.22 mm), with a few truncate setae. Ocelli large, forming almost equilateral triangle, POL:LOL:OOL = 4:3:8. Diameter of ocellus 3.5 × ommatidium diameter (0.07 mm/0.02 mm); malar space shorter than compound eye height (0.17 mm/0.30 mm) in lateral view. Occipital carina narrow and smooth in dorsal view. Palpal formula 5:2.

***Antennae*.** Antenna 12-segmented with nonabrupt clava, Al subcylindrical, broadened apically and reticulate sculptured. Size of antennomeres (length/width in millimeters) in lateral view: A1 0.51/0.11, A2 0.14/0.08, A3 0.19/0.07, A4 0.15/0.07, A5 0.14/0.08, A6 0.12/0.09, A7 0.15/0.11, A8 0.14/0.12, A9 0.15/0.13, A10 0.15/0.13, A11 0.14/0.14 and A12 0.21/0.14. Clavomeres A9–A11 subquadrate in dorsal view, A12 subconical, and all flattened ventrally. A1–A7 covered with truncate setae; A6–A12 covered with short pubescence.

***Mesosoma*** (Fig. 3D, E). Mesosoma at level of tegula wider than head (0.64 mm/0.60 mm). Cervix longitudinally strigose. Pronotal collar with dense pilosity. Mesoscutum slightly convex, smooth with sparse truncate setae. Anterior scutellar pit with three low carinae inside, subcircular, and deep. Mesoscutellar disc slightly convex; axilla as wide as scutellar pit (0.17 mm/ 0.15 mm), shortly pubescent. Metanotum covered with short pilosity and few truncate setae. Metascutellum armed with three longitudinal ridges; metanotal trough smooth. Propodeum reticulate rugose with scattered setae and postero-lateral corners strongly prominent and posterior margin deeply convex medially. Mesopleuron smooth with truncate, specialized setae. Metapleuron and lateral sides of propodeum rugose with truncate setae and covered with short pilosity. Forewing 3.15 × longer than wide (2.96 mm/0.94 mm); submarginal vein reaching proximal third of wing length. Hindwing longer than wide (1.87 mm/0.21 mm), with marginal vein. Legs long, covered with scattered truncate setae. Femora of all legs slender with long stalk.

***Metasoma*** (Fig. 3F, G). Petiole 1.47 × longer than wide (0.28 mm/0.19 mm), longitudinally rugulate, covered with long truncate setae and short pubescence. Gaster 2.05 × longer than wide (1.21 mm/0.59 mm), smooth, with few sparse truncate setae dorsally and ventrally.

***Variation*.** Anterior scutellar pit subcircular to remarkable “chestnut” form. Female body length 2.93–3.42 mm.

**Figure 5.**
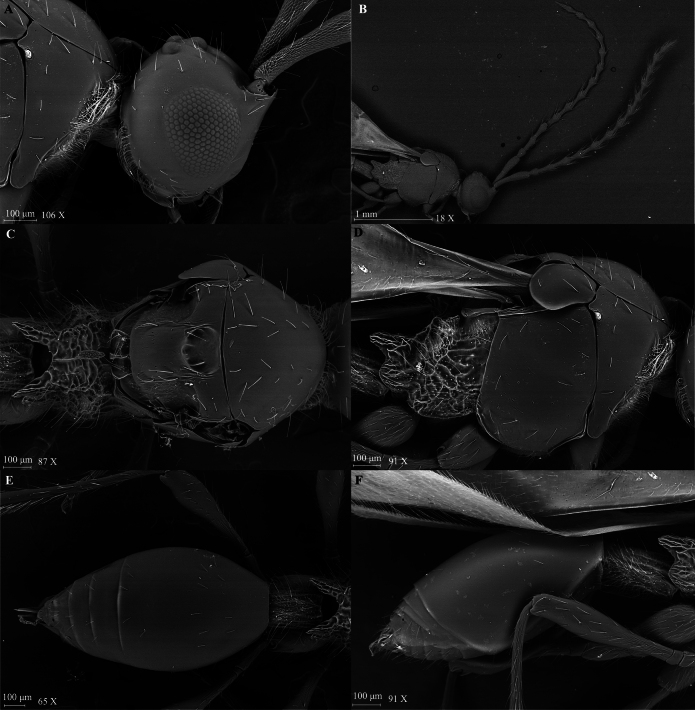
*Szelenyiopria fortunensis* sp. nov., male. A. Head in lateral view; B. Head and antennae in lateral view; C. Mesosoma in dorsal view; D. Mesosoma in lateral view; E. Metasoma in dorsal view; F. Metasoma in lateral view.

**Male** (Figs 4, 5A–F). Body length: 2.82–3.08 mm. Similar to female except for the following characters: eyes bare; antenna 14-segmented; A3–A14 each fusiform, its widest part slightly shifted towards apex with a row of long setae. Size of antennomeres (length/width in mm): A1 (0.54/0.1); A2 (0.15/0.1); A3 (0.2/0.08); A4 (0.2/0.09); A5 (0.2/0.09); A6 (0.23/0.08); A7 (0.19/0.09); A8 (0.2/0.09); A9 (0.19/0.09); A10 (0.16/0.08); A11 (0.15/0.08); A12 (0.18/0.08); A13 (0.16/0.08); A14 (0.29/0.07).

##### Distribution.

Known from only the type locality.

##### Biology.

*Szelenyiopria fortunensis* sp. nov. is a larval endoparasitoid of the leaf-cutter ant *Acromyrmex coronatus* in Panama. We found that 25% of all *A. coronatus* nests collected in Fortuna Forest Reserve were parasitized by this species. This new species can be solitary (only one wasp per larva) or gregarious (two or more wasps per larva), with each wasp separated from the others by a septum. The ant workers protected, groomed, and transported the parasitized larvae as if they were healthy larvae. However, after emergence the wasps tried to leave the nests quickly. If they did not, two or more worker ants used their mandibles to hold them by the legs, antennae, and/or wings, and then the wasps were mutilated and killed.

#### ﻿ Szelenyiopria chiriquiensis

Taxon classificationAnimaliaHymenopteraDiapriidae

Araúz & Fernández-Marín sp. nov.

0570AB70-4C44-5FA7-B383-8B378027417C

https://zoobank.org/4EC0006E-E74A-4CC7-8CE8-C1DEF7E07EF7

Figs 6, 7, 8, 9

##### Type material.

**Figure 6.**
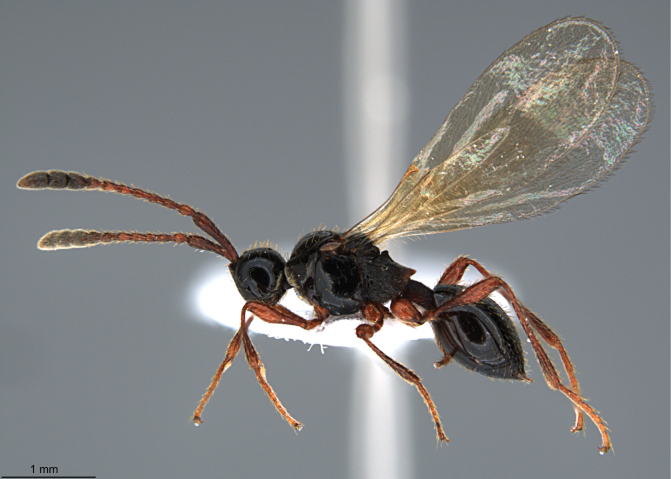
*Szelenyiopria chiriquiensis* sp. nov., holotype, female, habitus in lateral view.

***Holotype*** • female (Fig. 6); “PANAMÁ, Chiriquí, Boquerón, Cordillera (8°43'11.3"N, 82°35'52.4"W, 1200 m); 13.xii.2023. Emerged from a larva of *Acromyrmex coronatus* (nest #03), Gabriel Araúz, Hermógenes Fernández-Marín and Anel Pérez leg.” (MIUP). ***Paratypes*** • 3 males and 4 females, with same labels as holotype; • 5 females, PANAMÁ, Chiriquí, Tierras Altas, Volcán (8°48'41.2"N, 82°39'11.5"W); 13.xii.2023. Emerged from a larva of *A. coronatus* (nest #01), Gabriel Araúz, Hermógenes Fernández-Marín and Anel Pérez leg. Paratypes location: 6 females and 2 males in MIUP; 3 females and 1 male in LIS.

##### Etymology.

The species name is refers to term “Chiriquí”, which means “Valle de la Luna (Moon Valley)” to the Ngäbe and Buglé peoples, the native inhabitants of Chiriquí Province. Additionally, the species name refers to Chiriquí Province, where we discovered this species and which is one of the most beautiful, biodiverse, and agriculturally productive regions in Panama.

##### Diagnosis.

**Figure 7.**
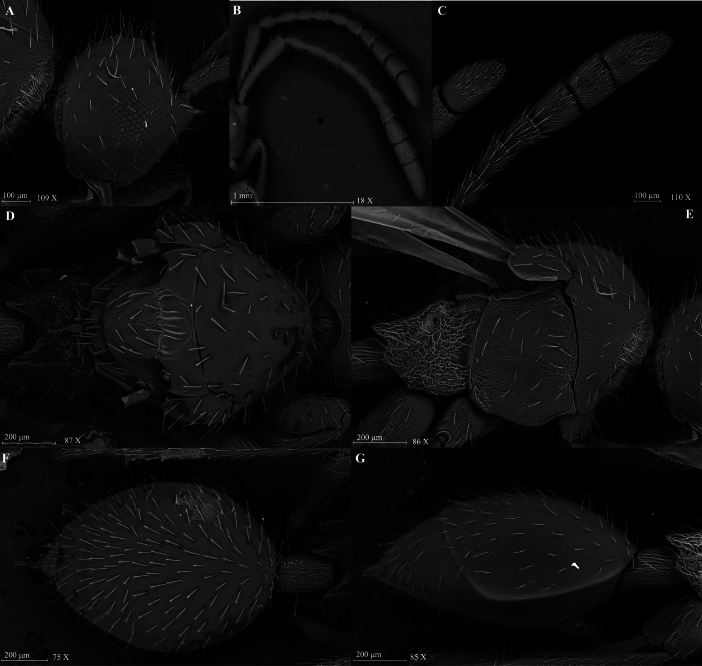
*Szelenyiopria chiriquiensis* sp. nov., female. A. Head, lateral view; B, C. Antennae in lateral view; D. Mesosoma in dorsal view; E. Mesosoma in lateral view; F. Metasoma in dorsal view; G. Metasoma in lateral view.

*Szelenyiopria chiriquiensis* differs from the all known species of the genus by the combination of the following characters: female antenna 11-segmented, with nonabrupt clava; three apical segments of clava flattened ventrally; gena with dense postgenal cushion; lateral side of pronotum and mesopleura mainly covered with alutaceous sculptured; anterior scutellar pit semicircle, with three low longitudinal striae and four ridges inside; mesoscutum convex and with alutaceous sculpture; propodeum dorsally rugulose sculptured with median keel slightly raised anteriorly (Figs 6, 7E); side of propodeum and metapleuron reticulate rugose, covered with scattered, apically truncate and up-standing setae and short pubescence. T2–T4 covered with numerous specialized setae (Fig. 7F, G). Male antenna 14-segmented; A3–A14 each with a row of long setae.

##### Description.

**Holotype, female** (Fig. 6). Body length 2.82 mm. Head, mesosoma and metasoma shiny, mesosoma nearly all covered with alutaceous sculpture. Head, mesosoma and metasoma black; antenna, tegula and legs pale brown.

***Head*** (Fig. 7A–C). Head subrectangular and slightly transverse, in dorsal view 1.29 × as wide as long (0.62 mm/0.48 mm) with protruding antennal shelf and covered with long, scattered, truncate setae. Frons and face smooth. Temple subequal to width of compound eye (0.22 mm/0.20 mm) in lateral view. Compound eyes ovoid, 1.05 × as high as wide (0.20 mm/0.19 mm), with some scattered truncate setae. Ocelli large, forming almost equilateral triangle, POL:LOL:OOL = 4:3:10. Ocelli diameter 4 × ommatidium diameter; malar space 0.55 × as long as compound eye height (0.11 mm/0.20 mm) in lateral view. Occipital carina narrow and smooth in dorsal view. Palpal formula 5:2.

***Antennae*.** Antenna 11-segmented with nonabrupt clava, A1 subcylindrical, broadened apically and covered with reticulate sculpture. Size of antennomeres (length/width in mm) in lateral view: A1 0.51/0.11, A2 0.13/0.08, A3 0.15/0.07, A4 0.14/0.07, A5 0.13/0.07, A6 0.13/0.07, A7 0.13/0.08, A8 0.13/0.09, A9 0.15/0.14, A10 0.16/0.15 and A11 0.28/0.15. Clavomeres A9–A10 subquadrate in dorsal view and flattened ventrally; A11 subconical, flattened ventrally. All antennomeres with long, scattered, truncate setae and A8–A11 covered with short pubescence.

***Mesosoma*** (Fig. 7D, E). Mesosoma 1.19 × wider than head at level of tegula (0.74 mm/0.62 mm). Cervix longitudinally strigose. Pronotal cushion dense, pronotal collar with only scattered truncate setae. Mesoscutum slightly convex, shiny, with alutaceous sculpture along margins and sparse truncate setae. Mesoscutellar disc convex and covered with alutaceous sculpture; axilla 1.33 × as long as the scutellar pit (0.18 mm/0.24 mm). Metanotum with alutaceous sculpture between longitudinal ridges on metascutellum and on metanotal trough, covered with short pilosity and scattered truncate setae. Metascutellum armed with three longitudinal ridges. Propodeum rugulose and covered by short pilosity, posterolateral corners strongly prominent and posterior margin deeply convex medially in dorsal view. Mesopleuron with scattered specialized setae and short pilosity, mainly covered with alutaceous sculpture and partly smooth in its middle part. Propleuron with alutaceous sculpture and covered with short and dense pilosity. Metapleuron and lateral sides of propodeum rugose, covered with short pale pilosity. Forewing 3.05 × longer than wide (2.78 mm/0.91 mm); submarginal vein reaching proximal third of forewing length. Hindwing longer than wide (1.69 mm/0.21 mm), with marginal vein. Legs long, covered with scattered truncate setae. Femora of all legs slender with long stalk.

***Metasoma*** (Fig. 7F, G). Petiole 1.47 × longer than wide (0.28 mm/0.19 mm), longitudinally strigose and covered with short pilosity. Gaster 1.44 × longer than wide (1.01 mm/0.7 mm) with dorsally numerous scattered, long, truncate setae and ventrally with two rows of long, truncate setae.

***Variation*.** Mesoscutum in some parts up to completely alutaceously sculptured. Female body length 3.05–3.10 mm.

**Figure 8.**
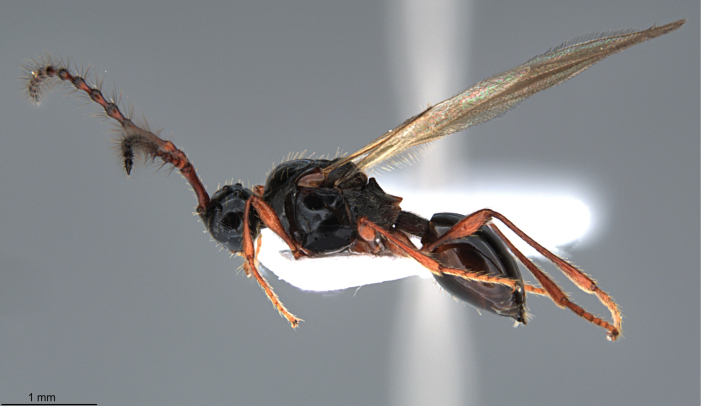
*Szelenyiopria chiriquiensis* sp. nov., paratype, male, habitus in lateral view.

**Figure 9.**
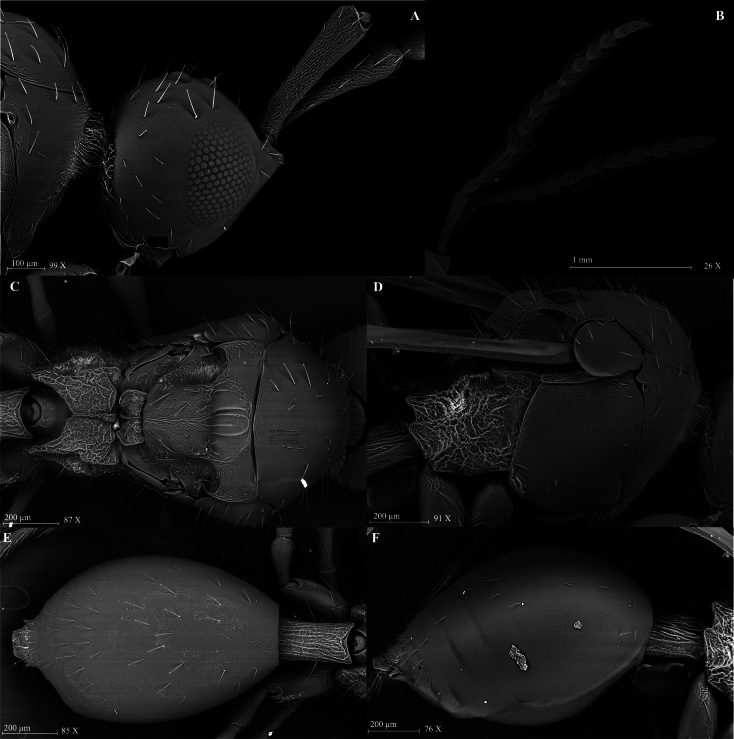
*Szelenyiopria chiriquiensis* sp. nov., male. A. Head, lateral view; B. Antennae in lateral view; C mesosoma in dorsal view; D. Mesosoma in lateral view; E. Metasoma in dorsal view; F. Metasoma in lateral view.

**Male** (Figs 8, 9A–F). Body length 3.38–3.43 mm. Similar to female except for the following characters: eyes bare; antennae 14-segmented; A1 and A2 subcylindrical, shortly pubescent; A3–A14 each fusiform, its widest part slightly shifted towards apex with a row of long setae. Size of antennomeres (length/width in mm): A1 (0.52/0.12); A2 (0.10/0.10); A3 (0.18/0.09); A4(0.20/0.10); A5 (0.20/0.09); A6 (0.18/0.10); A7 (0.22/0.10); A8 (0.23/0.10); A9 (0.21/0.09); A10 (0.22/0.10); A11 (0.20/0.10); A12 (0.19/0.09); A13 (0.18/0.09) and A14 (0.22/0.07).

##### Distribution.

Known from only the type locality.

##### Biology.

*Szelenyiopria chiriquiensis* sp. nov. is a larval endoparasitoid of the leaf-cutter ant *Acromyrmex coronatus* in Panama. We found that 62% of all *A. coronatus* nests collected were parasitized (*n* = 21). This diapriid species can be solitary (only one wasp per larva) or gregarious (up to five wasps per larva), with each wasp separated from the others by a septum. The ant workers protected, groomed, and transported the parasitized larvae as if they were healthy larvae. Once the wasps emerged, they tried to leave the nest quickly. If they did not, two or more ants used their mouthparts to hold them by the legs, antennae, and/or wings, and they dismembered the wasps.

### ﻿Key to female specimens of *Szelenyiopria*, adapted and expanded from Loiácono and Margaría (2000)

We expanded the key to female *Szelenyiopria* species provided by Loiácono and Margaría (2000) with the two new species of this study and another four species: *S. reichenspergeri* Ferrière, *S. talitae* Loiácono & Margaría; *S. loiaconae* Comério, Oliveira, Perioto & Lara; *S. jataiensis* Comério, Oliveira, Perioto & Lara. Illustrations of these species are available in their original descriptions, except *S. fortunensis* sp. nov., and *S. chiriquiensis* sp. nov., which are illustrated in this work.

**Table:** 

1	Antenna 12-segmented	**2**
–	Antenna 11-segmented	**4**
2	Antennal clava distinctly abrupt, 5-segmented, with A8–A11 distinctly transverse and flattened ventrally; A6–A7 as long as wide to weakly transverse; gena with scanty postgenal cushion; anterior scutellar pit rectangular	***S. loiaconae* Comério, Oliveira, Perioto & Lara**
–	Antennal clava 3-segmented, if >3 then clava nonabrupt, A8–A11 elongate as long as wide and A8 not flattened ventrally; A6–A7 distinctly elongate, gena with dense postgenal cushion; anterior scutellar pit subcircular	**3**
3	Antennal clava strongly abrupt 3-segmented, A9–A10 not flattened ventrally; anterior scutellar pit smooth inside; axilla and metapleuron with dense pilosity; petiole as long as wide	***S. jataiensis* Comério, Oliveira, Perioto & Lara**
–	Antennal clava nonabrupt, A9–A10 flattened ventrally; anterior scutellar pit with three low carinae inside; axilla and metapleuron with sparce pilosity; petiole elongate	***S. fortunensis* Araúz & Fernández-Marín, sp. nov.**
4	Antennal clava 3-segmented, A9 and A10 elongate; gena with short pilosity; anterior scutellar pit semicircular with three longitudinal carinae inside	***S. chiriquiensis* Araúz & Fernández-Marín, sp. nov.**
–	Antennal clava 4-segmented, A9–A10 more or less transverse; gena with dense cushion; anterior scutellar pit subcircular or subquadrate and smooth inside	5
5	Petiole as long as wide; head and mesosoma mat and predominantly alutaceous sculptured; anterior scutellar pit subrectangular and deep	***S. reichenspergeri* Ferrière**
–	Petiole elongate; head and mesosoma shiny, with fine rugulosity, if matte then coriaceous sculptured, anterior scutellar pit subcircular or subquadrate to slightly transverse and shallow	**6**
6	Anterior scutellar pit deep and slightly transverse; scutellar disc moderately convex, median keel moderately produced anteriorly	***S. talitae* Loiácono & Margaría**
–	Anterior scutellar pit shallow and subcircular or subquadrate, scutellar disc slightly convex or very convex, if slightly convex then median keel conspicuous	**7**
7	Head and mesosoma predominantly coriaceous; scutellum smooth; legs thick.	***S. coriacea* Loiácono**
–	Head and mesosoma predominately smooth and shiny; scutellum smooth or with striae and/or carina; legs slender	**8**
8	Gaster about twice as long as broad; scutellar shield with median carina and parallel longitudinal striae	***S. elongata* Loiácono**
–	Gaster about 1.4–1.7 × as long as broad; scutellar shield smooth or with median carina and oblique striae or only oblique striae beside the scutellar pit	**9**
9	Mesoscutum very convex; antennal shelf protuding; scutellar pit subcircular and deep, scutellar shield with median carina and oblique striae beside the scutellar pit; anterior half of the median keel of propodeum ligulate	***S. distinguenda* Loiácono**
–	Mesoscutum slightly convex; other features not entirely as above	10
10	Scutellar shield with some striae beside the scutellar pit; antennal shelf protruding; anterior half of the median keel of propodeum arrow-shaped	***S. clavata* Loiácono**
–	Scutellar shield smooth; antennal shelf only slightly protruding; anterior half of the median keel of propodeum ligulate or subcircular	11
11	Body with long, dense pilosity	***S. pilosa* Loiácono**
–	Body with scattered setae	12
12	Mesosoma lighter than metasoma; antennal club slightly distinct; gena and pronotal collar with very dense pilosity (Loiácono and Margaría 2000: fig. 31)	***S. masneri* Loiácono**
–	Mesosoma and metasoma dark chestnut brown, antennal club slightly to very distinct, gena and pronotal collar with dense pilosity (Loiácono and Margaría 2000: fig. 33)	**13**
13	Antennal club slightly distinct	***S. lucens* Loiácono**
–	Antennal club moderately to very distinct	**14**
14	Antennal shelf slightly protruding; scutellar lateral areas wide, anterior half of the median keel of propodeum subcircular	***S. minima* Loiácono**
–	Antennal shelf protruding; scutellar lateral areas narrow, anterior half of the median keel of propodeum ligulate	***S. pampeana* Loiácono**

### ﻿Geographic distribution

**Figure 10.**
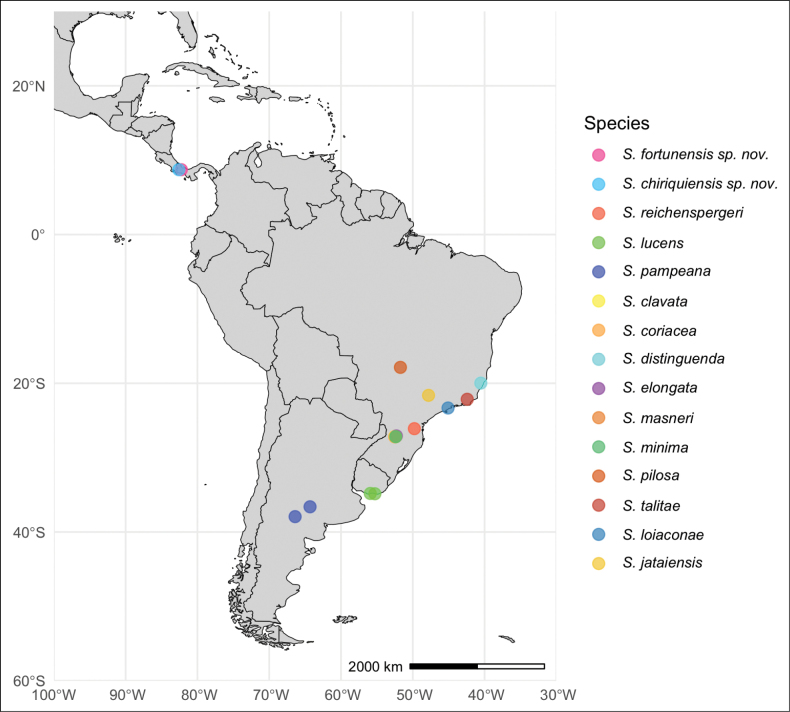
Distribution range of all 15 *Szelenyiopria* species, including the *S. fortunensis* sp. nov. and the *S. chiriquiensis* sp. nov.

Thirteen of the 15 *Szelenyiopria* species occur in South America, specifically in Argentina, Brazil, and Uruguay, while *S. fortunensis* sp. nov. and *S. chiriquiensis* sp. nov. are distributed in the mountains of western Panama (Fig. 10). Masner and García (2002) indicated that *Szelenyiopria* occurs from Guatemala to Argentina, but prior to our publication no species had been described for Central America.

### ﻿Parasitism rates and demography

**Figure 11.**
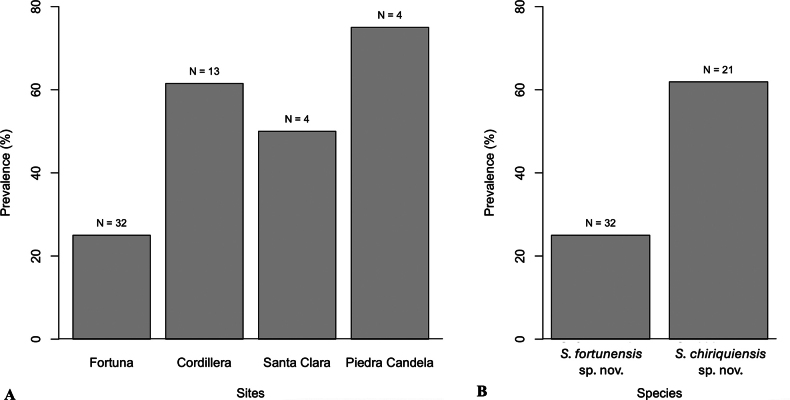
Parasitism prevalence rates by *Szelenyiopria* spp. in *Acromyrmex coronatus* nests. A. Prevalence by sites; B. Prevalence by wasp species. Numbers above the bars represent the total of nests collected.

The overall prevalence of both *Szelenyiopria* species in colonies of *A. coronatus* was 39.6% (*n* = 53, Table 1), which is highest in Piedra Candela (75.0%, *n* = 4) and lowest in Fortuna Forest Reserve (25.0%, *n* = 32) (Fig. 11A). The prevalence of the parasitoids by species was higher in *S. chiriquiensis* sp. nov. (61.9%) in comparison with *S. fortunensis* sp. nov. (25.0%, Fig. 11B).

**Table 1.** Prevalence of *Szelenyiopria fortunensis* sp. nov. and *S. chiriquiensis* sp. nov. that parasitized *A. coronatus* nests in Chiriquí, Panamá.

Year	*S. fortunensis* sp. nov.		*S. chiriquiensis* sp. nov.	
	Collected/parasitized nests	Prevalence %	Collected/parasitized nests	Prevalence (%)
2012	30/7	23.3%	—	—
2021	2/1	50%	4/2	50.0
2022	—	—	10/7	70.0
2023	—	—	7/4	57.1
Total	32/8	25%	21/13	61.9%

**Figure 12.**
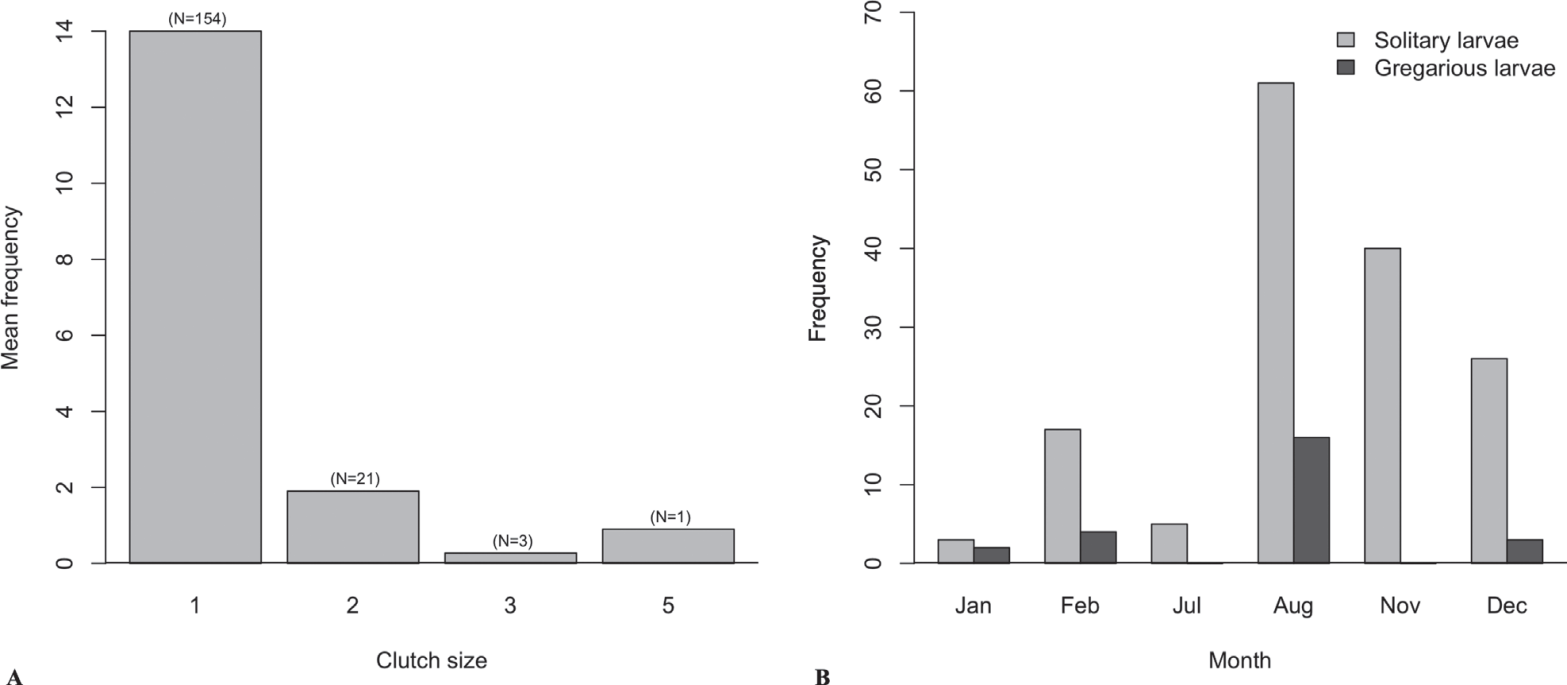
Clutch size of *S. chiriquiensis* sp. nov. in larvae of *Acromyrmex coronatus* in 11 nests. A. Mean frequency of clutch size per larvae; numbers in parentheses indicate the number of individuals found; B. Frequency of clutch size of single and multiple parasitism per month.

In total, 17 *A. coronatus* colonies contained 0–1 queen (0.35 ± 0.49), 0–9471 eggs (1852 ± 2310), 259–16933 larvae (3527 ± 3976), 115–13620 pupae (3168 ± 3280), and 6673–64449 workers (16885 ± 13050, Suppl. material 1: table S1). The intensity of the parasitoid of *S. chiriquiensis* sp. nov. ranged from 0.01% to 2.59%, and intensity mean per site was 0.56% in Cordillera to 1.21% in Piedra Candela (Suppl. material 1: table S2). The number of solitary larvae of *S. chiriquiensis* sp. nov. (1 individual per larva) was greater (*n* = 154) than the gregarious larvae (2–5 individuals per larvae) (*n* = 25) (Fig. 12A). Solitary parasitism was higher in August (38.5 ± 31.8), November (20.0 ± 28), and December (14.5 ± 16.3) than the rest of the year (Fig. 12B). More male wasps emerged than female wasps, in a 3.31:1.00 ratio (Suppl. material 1: table S3). The number of larvae parasitized was not influenced by the total number of ants (χ2 = 0.003, df = 15, p = 0.96), season (χ2 = 1.12, d.f. = 14, *p* = 0.23) or site (χ2 = 0.004, df = 13, *p* = 0.95). The number of parasitized larvae per colony tended to increase with the number of eggs per colony (χ2 = 8.1, df = 12, *p* = 0.004). However, this relationship changes if we remove an outlier colony (77 parasitized larvae), then the number of larvae parasitized tended to decrease with increases in the number of eggs (χ2 = 4.96, df = 11, *p* = 0.03).

## ﻿Discussion

Our study provides details on the parasite–host interactions of the fungus-growing ant *Acromyrmex coronatus* and diapriids wasps of the genus *Szelenyiopria* in Panama. *Acromyrmex coronatus* is the most widely distributed species of the genus, extending from Mexico to Argentina (AntWeb 2024), and the one with the most individuals per colony (Wetterer 1995). In Panama, *A. coronatus* is the only one of the five species of *Acromyrmex* that has been found with parasitoid wasps (HFM unpublished data), which suggests that their superficial nests on the soil surface or tree trunks may be easier to parasitize as compared with other *Acromyrmex* species that build nests deeper in the ground. Additionally, the broad distribution and high density of nests may make them a more attractive niche for parasitoid wasps (Fernández-Marín et al. 2006; Quevillon 2018; Almeida et al. 2021).

The prevalence of *Szelenyiopria fortunensis* sp. nov. and *S. chiriquiensis* sp. nov. species in *A. coronatus* nests was higher (39.6%) than the prevalence of *S. talitae* in *A. subterraneus* (22.0%) (Mattoso et al. 2021) and lower than the prevalence of *Mimopria* sp. (Diapriidae) in *A. landolti* nests (45.0%) (Rodrigues et al. 2015). Moreover, the intensity of parasitism by *S. chiriquiensis* sp. nov. was considerably lower (2.6%) in comparison to the intensity of parasitism of *S. talitae* on *A. subterraneus* (55.0–83.0%) (Mattoso et al. 2021), and of *Mimopria* sp. on *A. landolti* (12.0–20.1%) (Rodrigues et al. 2015).

Ant larvae parasitized by a single wasp larva were more frequent than those with multiple wasp larvae in our study. It is possibly because our collections were made before the appearance of sexual ant larvae, which develop into alate males and females. Sexual ant larvae appear between May and June and are larger in size and can host a higher number of koinobionts per larva than non-sexual smaller larvae. Thus, although our evidence is scarce, there is the possibility that during periods without sexual larvae, wasps place one egg per worker larvae, resulting in solitary parasitic larvae. However, it is possible that as sexual larvae become more prevalent, wasps will favor them, thereby increasing their reproductive fitness and reducing their risk of being attacked by ants, as has been observed in other parasitoid wasps (Liu et al. 2011). Future investigations should focus on multifactorial analyses, integrating host quality, population structure, and maternal physiological state, to unravel the mechanisms underlying sex ratio patterns.

The mechanisms that drive solitary or gregarious parasitism in diapriid wasps are not known. Individuals of *Szelenyiopria* from a single host have developmental patterns, melanization, and sclerotization in the pupal state that are not uniform (Suppl. material 2). Less developed individuals have whitish or translucent structures and short wings, while more developed ones have fully formed wings and dark coloration, indicating melanization and sclerotization, as a sign of differential development. We propose two non-exclusive hypotheses: multiple parasitism, whereby one or several adult wasp females parasitise a host larva by depositing multiple eggs, or polyembryony, whereby a single egg develops a number of clones through undetermined asexual processes (Segoli et al. 2010). Several individuals develop in a single host, and this can generate competition for both the food resource and available space, which can favor the success of few individuals (Segoli et al. 2010).

*Acromyrmex coronatus* workers cared for, cleaned, and transported parasitized larvae in the same way as they do with healthy larvae. However, upon emerging, the adult wasps are aggressively attacked by the ants and grab them, then cut off their wings, antennae, and legs. This behavior has been observed in all studies where the development of wasps in their hosts has been reported, including colonies of *Cyphomyrmex minutus* / *C. rimosus* (Fernández-Marín et al. 2006), *Trachymyrmex zeteki* (Peréz-Ortega et al. 2010), and *Apterostigma dentigerum* (González et al. 2016). With other parasitoids, this behavior has been observed in *Apterostigma dentigerum* ants that care for larvae parasitized by the ectoparasitoid fly *Pseudogaurax paratolmos* (Chloropidae), and workers attack adult flies after emergence (González et al. 2016) or remove them from the nest (Fernández-Marín et al. 2006; Loiácono and Margaría 2009).

Our study increases the number of known species of diapriid wasps in the Neotropics and provides basic information on the host-parasite interaction of parasitoid wasps and fungus-growing ants. Further research is required to investigate the behavioral and chemical mechanisms used by diapriid wasps to locate, identify, and parasitize ant larvae. This should include an examination of how the wasps enter the nest and oviposit larvae, and how they initially escape detection within the nest. Finally, *S. chiriquiensis* sp. nov. and *S. fortunensis* sp, nov. may be potential biological control agents of *A. coronatus*.

## Supplementary Material

XML Treatment for Szelenyiopria fortunensis

XML Treatment for Szelenyiopria chiriquiensis

## ﻿Additional information

### Use of AI

No use of AI was reported.

### Author ORCIDs

Vasilisa Chemyreva https://orcid.org/0000-0002-6547-6259

Tomás Ríos https://orcid.org/0000-0003-0590-6488

Dumas Gálvez https://orcid.org/0000-0001-8699-8497

Roberto A. Cambra https://orcid.org/0000-0002-3799-4710

### Data availability

All of the data that support the findings of this study are available in the main text or Supplementary Information.

## Supplementary materials

Supplementary material 1 Supplementary tables Data type docx https://binary.pensoft.net/file/1406832This dataset is made available under the Open Database License (http://opendatacommons.org/licenses/odbl/1.0/). The Open Database License (ODbL) is a license agreement intended to allow users to freely share, modify, and use this Dataset while maintaining this same freedom for others, provided that the original source and author(s) are credited.Gabriel Araúz, Vasilisa Chemyreva, Tomás Ríos, Dumas Gálvez, Roberto A. Cambra, Enrique Medianero, William T. Wcislo, Hermógenes Fernández-Marín

Supplementary material 2 Supplementary image Data type png https://binary.pensoft.net/file/1406833This dataset is made available under the Open Database License (http://opendatacommons.org/licenses/odbl/1.0/). The Open Database License (ODbL) is a license agreement intended to allow users to freely share, modify, and use this Dataset while maintaining this same freedom for others, provided that the original source and author(s) are credited.Gabriel Araúz, Vasilisa Chemyreva, Tomás Ríos, Dumas Gálvez, Roberto A. Cambra, Enrique Medianero, William T. Wcislo, Hermógenes Fernández-Marín
