# Supplementary material for: ﻿Two new species of Szelenyiopria Fabritius (Hymenoptera, Diapriidae), larval koinobiont endoparasitoids of the leaf-cutter ant Acromyrmex coronatus (Fabricius) (Hymenoptera, Formicidae), from Panama
Source: Zookeys. 2025 Sep 1;1250:293–314. doi: 10.3897/zookeys.1250.151740 (PMC12418030; doi:10.3897/zookeys.1250.151740)
Supplement: Supplementary material 1 — Supplementary tables [file zookeys-1250-293_article-151740__-s001.docx]

**Supporting material**

Table S1. Demography of 17 colonies of *Acromyrmex coronatus* in Chriquí, Panama.

| N° | Site | Queen | Eggs | Non parasitized  larvae | Parasitized larvae | Total larvae | Pupae | Workers | Total |
| --- | --- | --- | --- | --- | --- | --- | --- | --- | --- |
| 1 | Cordillera | 1 | 265 | 3158 | 21 | 3179 | 785 | 8377 | 12607 |
| 2 | Cordillera | 1 | 0 | 1723 | 2 | 1725 | 1932 | 14188 | 17846 |
| 3 | Cordillera | 0 | 236 | 1579 | 0 | 1579 | 2053 | 12833 | 16701 |
| 4 | Cordillera | 1 | 1981 | 2446 | 4 | 2450 | 3712 | 17217 | 25361 |
| 5 | Cordillera | 0 | 1289 | 438 | 1 | 439 | 216 | 12392 | 14336 |
| 6 | Cordillera | 1 | 1487 | 3105 | 0 | 3105 | 4629 | 10696 | 19918 |
| 7 | Cordillera | 1 | 9471 | 4884 | 77 | 4961 | 3473 | 6673 | 24579 |
| 8 | Cordillera | 0 | 805 | 256 | 3 | 259 | 115 | 21674 | 22853 |
| 9 | Cordillera | 0 | 1649 | 16918 | 15 | 16933 | 13620 | 64449 | 96651 |
| 10 | Cordillera | 0 | 2705 | 4259 | 0 | 4259 | 3376 | 15573 | 25913 |
| 11 | Cordillera | 0 | 4376 | 4786 | 0 | 4786 | 6376 | 13970 | 29508 |
| 12 | Cordillera | 0 | 1877 | 3361 | 0 | 3361 | 3965 | 16666 | 25869 |
| 13 | Cordillera | 0 | 324 | 8012 | 1 | 8013 | 2329 | 20431 | 31097 |
| 14 | Santa Clara | 0 | 563 | 1502 | 40 | 1542 | 1469 | 6973 | 10547 |
| 15 | Santa Clara | 0 | 411 | 1481 | 13 | 1494 | 5079 | 14387 | 21371 |
| 16 | Santa Clara | 0 | 620 | 870 | 0 | 870 | 196 | 9551 | 11237 |
| 17 | Santa Clara | 1 | 3426 | 1195 | 2 | 1197 | 538 | 17611 | 22773 |
| Total (mean±SD) | | 0.35±0.49 | 1852.1±2310.0 | 3527.8±3976.4 | 10.5±20.2 | 3538.4±3979.1 | 3168.4±3280.8 | 16685.9±13050 | 25245.1±19407.6 |

Table S2. Within-nest parasitism rates of 11 colonies of *Acromyrmex coronatus* in Chriquí, Panama.

| N° | Site | Queen | Eggs | Non parasitized larvae | Parasitized  larvae | Total larvae | Pupae | Workers | Total | Intensity  rates (%) |
| --- | --- | --- | --- | --- | --- | --- | --- | --- | --- | --- |
| 1 | Cordillera | 1 | 265 | 3158 | 21 | 3179 | 785 | 8377 | 12607 | 0.66 |
| 2 | Cordillera | 1 | 0 | 1723 | 2 | 1725 | 1932 | 14188 | 17846 | 0.12 |
| 3 | Cordillera | 1 | 1981 | 2446 | 4 | 2450 | 3712 | 17217 | 25361 | 0.16 |
| 4 | Cordillera | 0 | 1289 | 438 | 1 | 439 | 216 | 12392 | 14336 | 0.23 |
| 5 | Cordillera | 1 | 9471 | 4884 | 77 | 4961 | 3473 | 6673 | 24579 | 1.55 |
| 6 | Cordillera | 0 | 805 | 256 | 3 | 259 | 115 | 21674 | 22853 | 1.16 |
| 7 | Cordillera | 0 | 1649 | 16918 | 15 | 16933 | 13620 | 64449 | 96651 | 0.01 |
| 8 | Cordillera | 0 | 324 | 8012 | 1 | 8013 | 2329 | 20431 | 31097 | 0.09 |
| 9 | Piedra Candela | 0 | 563 | 1502 | 40 | 1542 | 1469 | 6973 | 10547 | 2.59 |
| 10 | Piedra Candela | 0 | 411 | 1481 | 13 | 1494 | 5079 | 14387 | 21371 | 0.87 |
| 11 | Piedra Candela | 1 | 3426 | 1195 | 2 | 1197 | 538 | 17611 | 22773 | 0.17 |

Table S3. Sex Ratio (female:male) of *Szelenyiopria* wasps emerged in 11 *Acromyrmex coronatus* colonies in Chiriquí, Panama.

| N° | ID Nest | Emerged wasps | Female wasps | Male wasps | Sex ratio (female:male) |
| --- | --- | --- | --- | --- | --- |
| 1 | 20220502-01Ac | 8 | 3 | 5 | 0.6:1 |
| 2 | 20220205-02Ac | 0 | 0 | 0 | 0 |
| 3 | 20223107-04Ac | 4 | 1 | 3 | 0.3:1 |
| 4 | 20223107-05Ac | 1 | 0 | 1 | 0:1 |
| 5 | 20220608-07Ac | 23 | 2 | 21 | 0.1:1 |
| 6 | 20232001-12Ac | 8 | 5 | 3 | 1.7:1 |
| 7 | 20231312-07Ac | 1 | 1 | 0 | 1:0 |
| 8 | 20231312-02Ac. | 0 | 0 | 0 | 0 |
| 9 | 20221111-08Ac | 1 | 0 | 1 | 0:1 |
| 10 | 20221612-09Ac | 0 | 0 | 0 | 0 |
| 11 | 20232001-11Ac | 10 | 1 | 9 | 0.1:1 |
