# Supplementary figures and images for: ﻿Two new species of Szelenyiopria Fabritius (Hymenoptera, Diapriidae), larval koinobiont endoparasitoids of the leaf-cutter ant Acromyrmex coronatus (Fabricius) (Hymenoptera, Formicidae), from Panama
Source: Zookeys. 2025 Sep 1;1250:293–314. doi: 10.3897/zookeys.1250.151740 (PMC12418030; doi:10.3897/zookeys.1250.151740)

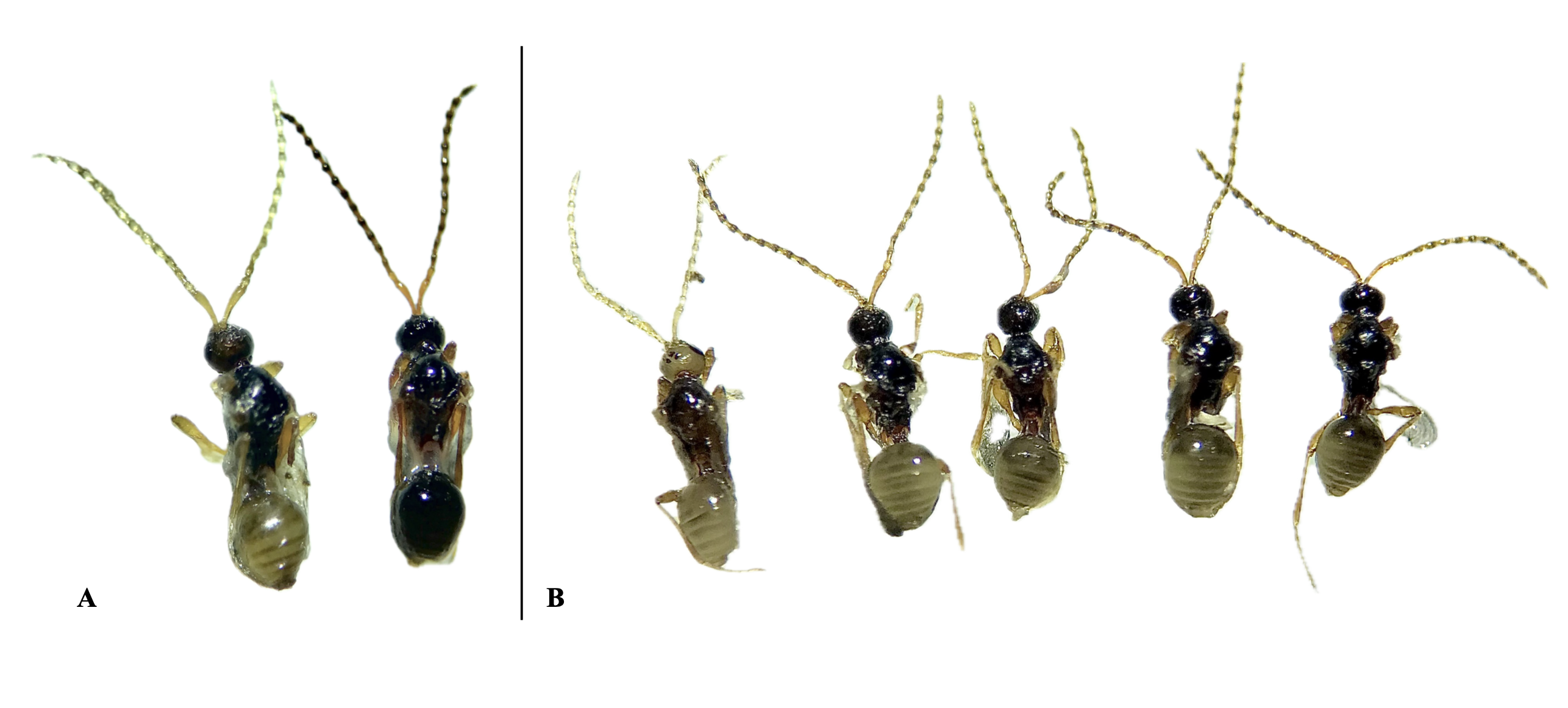

Supplement: Supplementary material 2 — Supplementary image [file zookeys-1250-293_article-151740__-s002.png]
